# Predicting Peritoneal Carcinomatosis in Locally Advanced Gastric Cancer: The Significance of Tumor Markers in the Peritoneal Washing

**DOI:** 10.1007/s12029-023-00984-7

**Published:** 2023-11-15

**Authors:** João Luís Pinheiro, Liliana Duarte, Andreia J. Santos, André Tojal, Carolina Canhoto, Marta Ferreira, Conceição Marques, Jorge Pereira

**Affiliations:** 1 General Surgery Department, Centro Hospitalar Tondela-Viseu, Avenida Rei Dom Duarte, 3504-509 Viseu, Portugal; 2 Faculty of Health Sciences, University of Beira Interior, Convento de Santo António, 6201-001 Covilha, Portugal

**Keywords:** Gastric cancer, Tumor markers, Peritoneal washing, Staging laparoscopy, Peritoneal carcinomatosis

## Abstract

**Purpose:**

Gastric cancer is the fifth most common malignant tumor worldwide. Many attempts have been made over the years to investigate the relationship between tumor markers and the risk of recurrence. This study aims to explore the predictive value of tumor markers measured in peritoneal washing during staging laparoscopy, regarding peritoneal carcinomatosis and mortality within 1 year.

**Methods:**

Patients with locally advanced gastric cancer, staged as at least usT2anyNM0 were submitted to staging laparoscopy in a Portuguese single center. CA 19.9, CEA, CA 125, and CA 72.4 were measured in the peritoneal washing after being harvested during staging laparoscopy.

**Results:**

Thirty-eight patients were enrolled. After 1 year, 20 patients did not recur (52.5%), 11 (28.9%) developed carcinomatosis, and 7 (18.4%) had distant metastasis. Mortality reached 23.7% (*n* = 9). A statistically significant prediction of carcinomatosis was obtained for CA 125 (cutoff: 107.6 U/mL (*p* = 0.019)) and CEA (cutoff: 2.0 ng/mL (*p* = 0.020)) with 87.5% and 75% sensitivity, respectively. Prediction of mortality was significant for CA 125 (cutoff: 103.8 U/mL (*p* = 0.044)) and CA 125 + CEA (*p* = 0.030). CEA and CA 125 had NPVs of 87.9% and 93.1% regarding PC, respectively. NPVs of 88.9% and 89.2% were met concerning mortality, for the same tumor markers.

**Conclusion:**

Performing the peritoneal liquid harvest during staging laparoscopy makes this analysis cost effective, reproducible, and does not add further morbidity. CA 125 and CEA, individually and in association, are good predictors of progression of disease and mortality within a year of staging laparoscopy in GC patients.

## Introduction

Gastric cancer (GC) is currently the fifth most common malignant tumor worldwide and remains one of the most frequent causes of cancer-related death [1, 2]. Despite the novel treatment strategies combining perioperative chemotherapy and surgery, the prognosis remains poor when diagnosed in advanced stages [3, 4].

To date, efforts to predict the progression of disease have been discouraging. Due to its rapid progression, early detection seems to be a key factor for the success of surgical treatment and improvement of overall survival in gastric cancer patients [4].

Many recognized prognostic indicators based on local and systemic extent of disease, such as the TNM system, offer important staging information taking into account clinical, radiologic and pathologic findings [5]. Staging laparoscopy (SL) has also been a part of the available diagnostic set of procedures that improve preoperative staging by detecting occult peritoneal disease in advanced stage tumors [6, 7]. As part of the SL, peritoneal washing cytology allows the detection of free cancer cells before any peritoneal deposits are macroscopically evident, thus potentially altering the treatment strategy [8].

In order to predict the course of disease, many attempts have been made over the years to investigate the relationship between tumor markers (TMs) and the risk of recurrence [9]. Because their serum measurement is commonly available and is relatively cheap, they pose an important monitoring tool to avoid undertreatment and improve follow-up efficacy [10].

Serum levels of carcinoembryonic antigen (CEA), carbohydrate antigen (CA) 19–9, and CA 72–4 have been associated with active digestive tract neoplasms including gastric cancer, and some have even been shown to predict its curability, particularly CA125 [3]. So far, some studies have tried to measure TMs in the peritoneal fluid in order to improve sensitivity of cytology when differentiating benign from malignant ascites [11–13]. However, the predictive value of peritoneal washing TMs has yet to be broadly studied. In the present study, we aim to investigate the prognostic and predictive value for peritoneal carcinomatosis (PC) of different TMs measured in the peritoneal washing during SL gastric cancer patients.

## Methods

### Patient Selection

In this prospective observational study, adult patients diagnosed with gastric adenocarcinoma in Centro Hospitalar Tondela-Viseu between February 2020 and March 2022 were enrolled and then selected based on the indication for SL. Subjects with locally advanced gastric cancer, staged as at least usT2anyNM0, were submitted to SL before undergoing any type of treatment. Exclusion criteria included Gastroesophageal Junction Siewert I and II Adenocarcinomas, peritoneal dissemination on SL, and cytotoxic systemic treatment done prior to the diagnosis for any other tumor. All enrolled patients had a peritoneal washing cytology negative for malignant cells.

The study design, selection criteria, and sample harvest were unanimously approved by our Institution’s Ethics Committee (Reference Number: 02/14/09/2020).

### Staging Laparoscopy and Method of Harvest

SL was performed using two 5 mm working ports and one 11 mm port for a 30° laparoscope inserted through the umbilicus. Pneumoperitoneum was kept at low intraabdominal pressures with carbon dioxide (CO_2_) at 12 mmHg. Every procedure involved assessment of the primary tumor and close inspection of all four quadrants of the peritoneal cavity in a clockwise fashion, including diaphragmatic domes, liver surface, teres and falciform ligaments, hepatic pedicle, omental bursa, paracolic recesses, colon, small intestine, mesentery, and pelvic cavity.

For the peritoneal washing harvest, 100 mL of saline was instilled in the peritoneal cavity and two samples were collected, with 50 mL each, for both cytology and TM assay. Samples were processed in an independent laboratory outside our institution. When the quantitative measurement of a TM was less than the limit of detection of the laboratory assay, the minimal detectable concentration was considered for the statistical analysis.

### Data Collection

Demographic data was collected regarding sex, age at diagnosis, and performance status according to the American Society of Anesthesiologists physical status score (ASA). The clinical staging (cTNM), histologic grade and pattern (World Health Organization Classification, 5th edition) [5], signet ring cell phenotype, and human epidermal growth factor receptor-type 2 (HER-2) status were recorded, along with the pathological staging (pTNM) after surgery, type of chemotherapy regimen, and number of cycles undergone. Regarding surgical procedure, all the patients were categorized according to the intraoperative findings. They were considered inoperable, submitted to radical resection or palliative surgery. The patients who were operated with curative intent underwent either open or laparoscopic radical D2 subtotal or total gastrectomy as described by the Japanese Gastric Cancer Association (5th Edition) [14].

One year after SL, peritoneal carcinomatosis was documented by routine follow-up imaging, either by computed tomography (CT) or positron emission tomography (PET). In the event of an urgent presentation or diagnosis of disease progression before the 1-year mark, the same imaging studies were considered for documentation of peritoneal disease.

### Statistical Analysis and Primary Endpoints

Statistical analyses were conducted using the Statistical Package for the Social Sciences (SPSS), version 23.0, and a value of *p* < 0.05 was regarded as statistically significant. The presented data was checked for normality using the Shapiro–Wilk test. The association between both serum and peritoneal washing TM assays and clinicopathological data with PC and mortality 1 year after SL was tested using chi-squared test (${\chi }^{2}$) and logistic regression models to adjust the outcomes for surgical treatment intent.

The predictive value of peritoneal washing TM measurements was studied using receiver-operated characteristic (ROC) curves. When more than one significant predictive marker was found, the same ROC analysis was performed to test for combined sensitivity and specificity.

The primary endpoint of the presented work is to study the predictive value of TMs measured in the peritoneal washing of staging laparoscopies in GC patients, regarding disease progression with PC or mortality after 1 year.

## Results

**Fig. 1:**
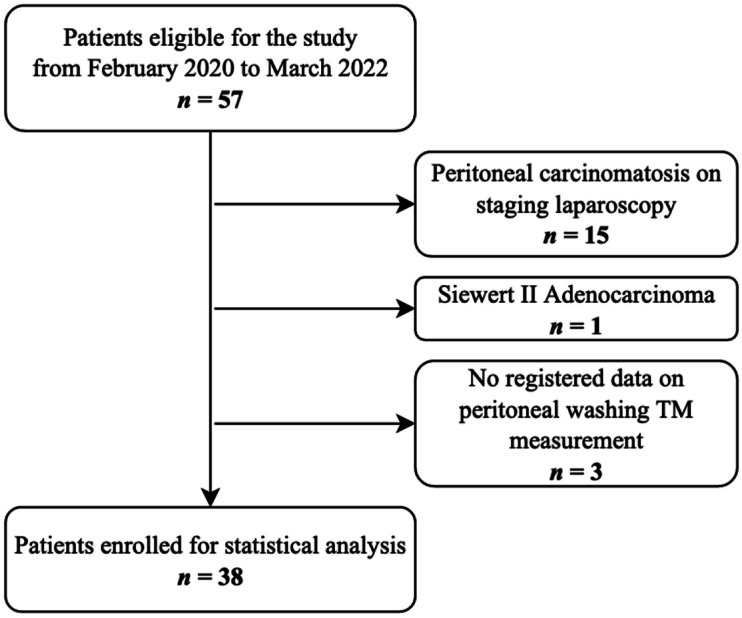
Flowchart of patient selection. TM, tumor marker

Patient selection and applied exclusion criteria are described in Fig. 1. From an initial pool of 57 patients submitted to SL, 15 had synchronous PC of which eight had a positive peritoneal washing cytology, three did not have TM measurements successfully recorded, and one patient was excluded from this analysis for being a Siewert II GEJ adenocarcinoma. A total of 38 patients were enrolled for TM quantification, follow-up and statistical analysis.

Clinicopathological data of the 38 patients are shown in Table 1. Median age of diagnosis was 70 years old, with 65.8% being male patients. ASA II status was the most frequent amongst the subjects, and the preferred staging method, along with abdominal, pelvic, and thoracic CT, was endoscopic ultrasound (EUS), performed in 78.9% of the study population. Most patients were diagnosed with locally advanced disease, with only 5.3% staged as cT2, and none as cT1. 89.5% of the patients had lymph node involvement, and the most common histologic subtype was the tubular pattern adenocarcinoma. Seven patients did not undergo any systemic treatment either because they refused (*n* = 1), or were not fit to withstand chemotherapy. The patients that were not submitted to surgery either refused surgical treatment (*n* = 1), had disease progression under neoadjuvant chemotherapy (*n* = 2), were deemed unfit for surgery during the course of chemotherapy (*n* = 1), or were unfit for both chemotherapy and surgery (*n* = 3). The used chemotherapy regimen was perioperative FLOT (fluorouracil plus leucovorin, oxaliplatin, and docetaxel) according to FLOT4 trial, except in one case where the patient underwent adjuvant chemotherapy with FOLFOX (fluorouracil plus leucovorin and oxaliplatin) [15]. All FLOT-receiving patients completed the planned eight cycles. Lymphadenectomy was performed as described by the Japanese Gastric Cancer Treatment Guidelines, following the current global standard [1, 14]. Out of the 23 patients operated with curative intent, two had D1+ lymph node dissection done and the remainder 21 underwent D2 lymphadenectomy.

**Table 1:** Clinicopathological data of the 38 patients enrolled in the study and outcome one year after staging laparoscopy

Data	*n* (%)
Sex	
Male	25 (65.8)
Female	13 (34.2)
Age at diagnosis (median, range)	70, 56–91
ASA status	
II	13 (34.2)
III	24 (63.2)
IV	1 (2.6)
Staging method	
EUS + CT	30 (78.9)
CT	8 (21.1)
cT	
2	2 (5.3)
3	24 (63.2)
4a	8 (21.1)
4b	4 (10.5)
cN	
N0	4 (10.5)
N+	34 (89.5)
Histologic pattern	
Undifferentiated	1 (2.6)
Mixed type	10 (26.3)
Mucinous	1 (2.6)
Poorly cohesive	5 (13.2)
Tubular	20 (52.6)
Tubulopapilar	1 (2.6)
Signet-ring variant	
Present	5 (13.2)
Absent	32 (84.2)
HER-2 status	
Positive	2 (5.3)
Negative	29 (76.3)
Missing data	7 (18.4)
Perioperative or adjuvant chemotherapy	
Yes	31 (81.6)
No	7 (18.4)
Surgical approach	
Laparoscopic	11 (28.9)
Open	20 (52.6)
Not applicable	7 (18.4)
Surgical treatment intent	
No surgery	7 (18.4)
Radical resection	23 (60.5)
Palliative procedure	8 (21.1)
One-year status	
No recurrence documented	20 (52.5)
Peritoneal carcinomatosis	11 (28.9)
Other type of recurrence	7 (18.4)
Dead	9 (23.7)

* EUS* endoscopic ultrasound, *CT* Computed Tomography

One year after SL, 20 patients did not have any type of recurrence (52.5%), 11 (28.9%) developed peritoneal implants, and seven patients (18.4%) had distant metastasis diagnosed on follow-up CT. After 1 year, mortality reached 23.7% (*n* = 9).

The association between clinical data, peritoneal carcinomatosis, and mortality 1 year after SL is summarized in Table 2. A significant correlation was found at 5% significance level between cT status and PC (*p* = 0.022), cT status and mortality (*p* = 0.032), and histologic grade and mortality (*p* = 0.04). Surgical treatment intent was associated with both outcomes, “no surgery” positively correlated with PC (OR 2.5, *p* = 0.008), and palliative surgery strongly associated with 1-year mortality (OR 22.0, *p* = 0.013) in logistic regression models.

**Table 2:** Results of the univariate analysis regarding carcinomatosis and mortality, 1 year after staging laparoscopy

	Peritoneal carcinomatosis		Mortality	
	Chi-square test	*p* value	Chi-square test	*p* value
Gender	0.032	0.858	0.549	0.459
cT	7.613	0.022	6.879	0.032
cN	1.821	0.177	1.387	0.239
Histology	7.613	0.179	8.124	0.150
Grade	5.566	0.062	6.446	0.040
ASA status	0.850	0.654	3.386	0.184
Surgical treatment intent	12.452	0.002	12.158	0.002
Surgical approach	10.634	0.005	5.782	0.056
Systemic treatment	21.170	0.004	16.635	0.020

The serum and peritoneal TMs were individually compared, and a statistically significant correlation was found between serum and peritoneal CA 19.9 (*p* = 0.001) and CA 72.4 (*p* = 0.049). The other tested TMs did not show a significant association (CEA, *p* = 0.148; CA 125, *p* = 0.416).

**Fig. 2:**
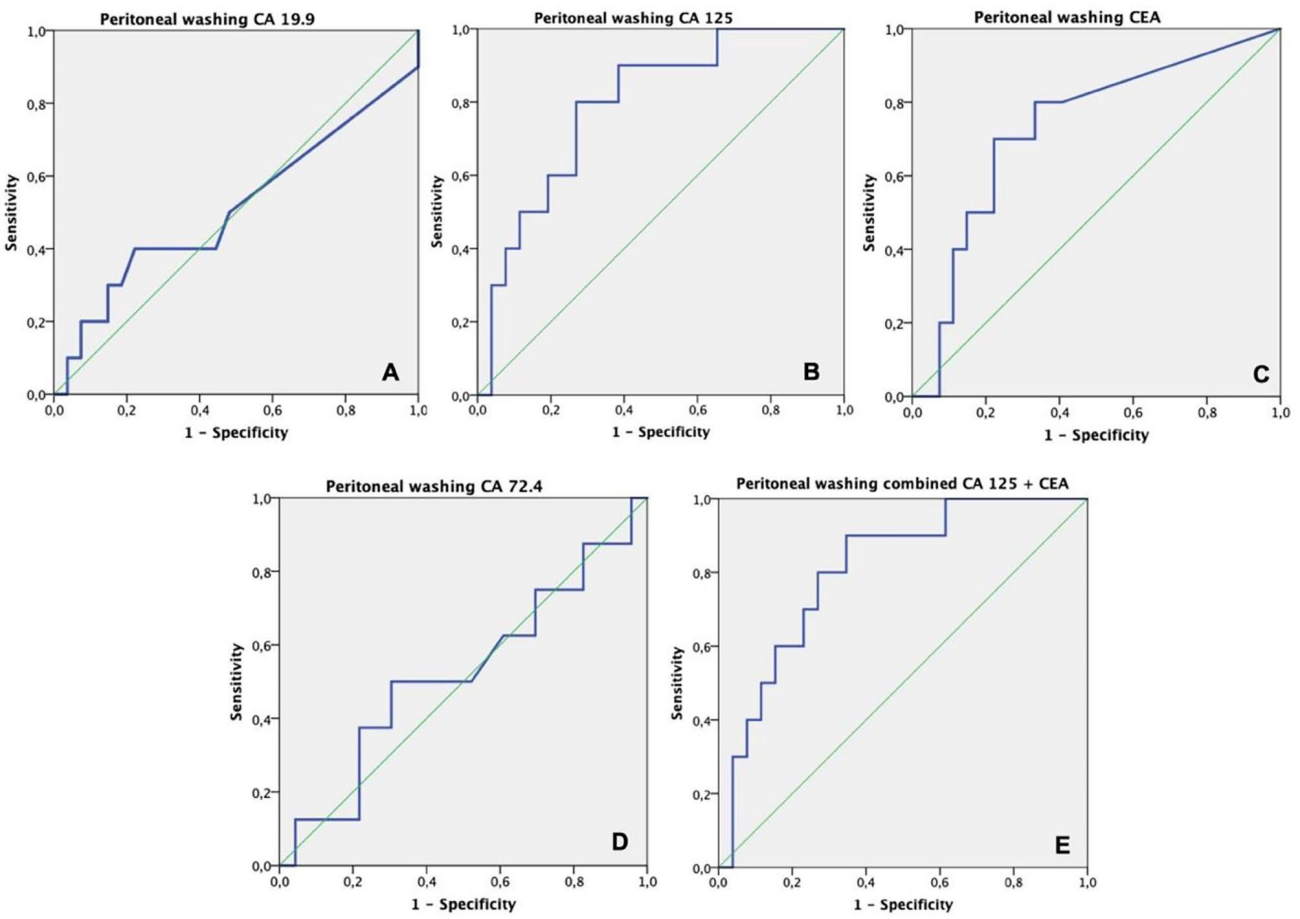
Graph representation (ROC curve) of the predictive performance of isolated tumor markers measured during staging laparoscopy in the peritoneal washing regarding peritoneal carcinomatosis after 1 year; **A** CA 19.9 (area = 0.51); **B** CA 125 (area = 0.79); **C** CEA (area = 0.73); **D** CA 72.4 (area = 0.52); **E** combined performance of CA 125 and CEA (area = 0.80)

**Fig. 3:**
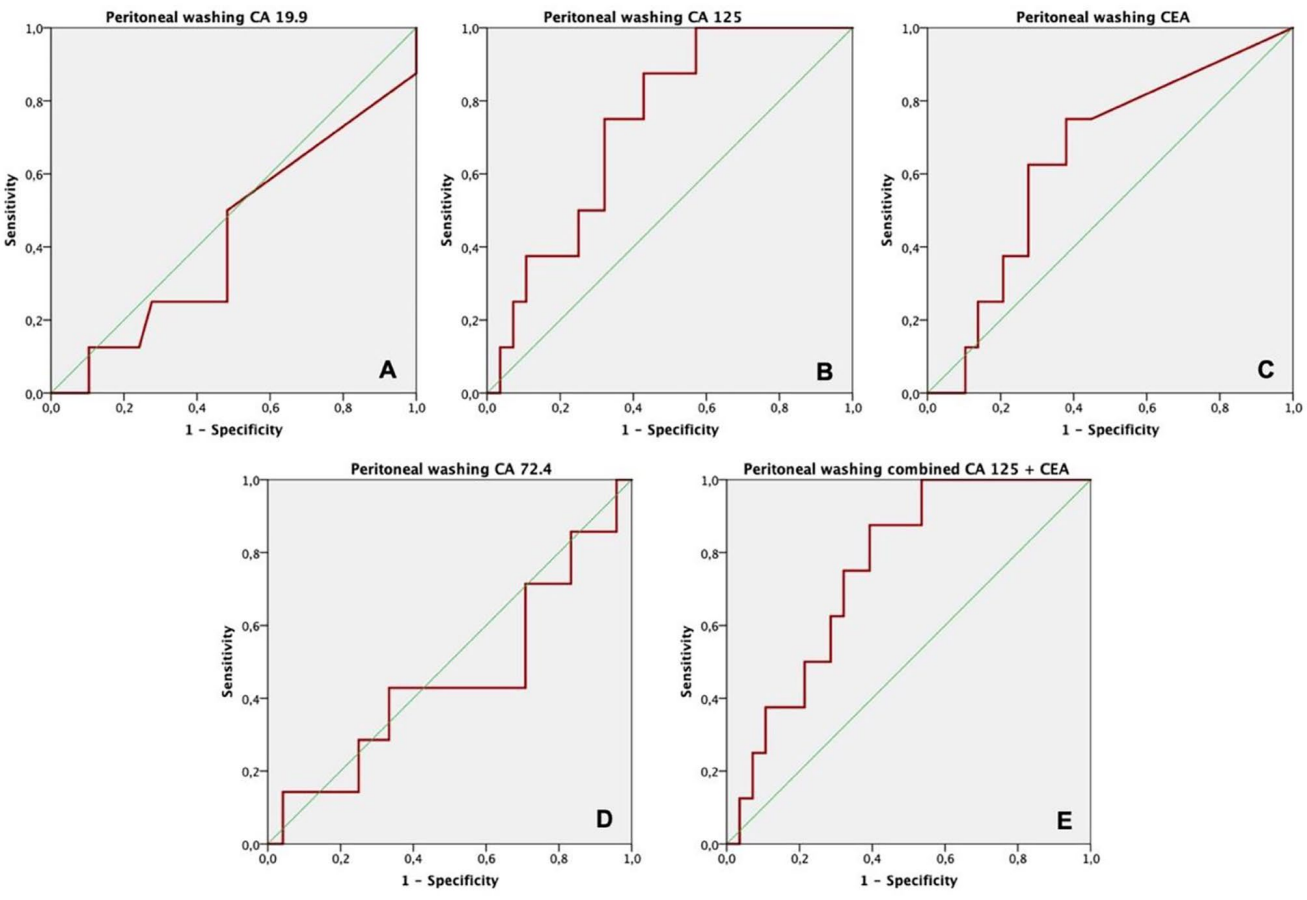
Graph representation (ROC curve) of the predictive performance of isolated tumor markers measured during staging laparoscopy in the peritoneal washing regarding mortality after one year. **A** CA 19.9 (area = 0.43); **B** CA 125 (area = 0.73); **C** CEA (area = 0.64); **D** CA 72.4 (area = 0.45); **E** combined performance of CA 125 and CEA (area = 0.75)

Regarding peritoneal washing TM assays, a ROC analysis (Table 3) was conducted to test their isolated predictive performance in terms of progression of disease with PC (Fig. 2) and mortality (Fig. 3) 1 year after SL.

**Table 3:** Performance of different tumor markers on predicting peritoneal carcinomatosis and mortality 1 year after staging laparoscopy

Tumor marker	Cutoff value	AUC	*p* value	Sensitivity (%)	Specificity (%)	PPV (%)	NPV (%)	95% CI of AUC	
								Lower bound	Upper bound
Prediction of peritoneal carcinomatosis									
CA 19.9	4.2	0.463	0.760	37.5	72.7	-	-	0.200	0.726
CA 125	107.6	0.784	0.019	87.5	69	53.4	93.1	0.607	0.961
CEA	2.0	0.781	0.020	75	72.7	52.8	87.7	0.593	0.969
CA 72.4	2.45	0.523	0.851	50	68.2	-	-	0.279	0.766
CA125 + CEA	-	0.801	0.013	87	68.2	52.7	92.8	0.633	0.969
Prediction of mortality									
CA 19.9	1.3	0.431	0.555	50	51.7	-	-	0.202	0.660
CA 125	103.8	0.737	0.044	75	64.3	39.5	89.2	0.567	0.907
CEA	1.15	0.647	0.210	75	62.1	38.1	88.9	0.440	0.853
CA 72.4	2.3	0.452	0.705	42.9	62.5	-	-	0.192	0.712
CA125 + CEA	-	0.754	0.030	75	60.7	37.2	88.7	0.592	0.917

*PPV* positive predictive value, *NPV* negative predictive value, *AUC* area under the curve, *CI* confidence interval

A statistically significant prediction of PC was obtained when plotted for CA 125 for a cutoff value of 107.6 U/mL (*p* = 0.019, 95% CI) and CEA for a cutoff value of 2.0 ng/mL (*p* = 0.020, 95% CI) with 87.5% and 75% sensitivity, respectively. When used in association, their joined predictive power remained statistically significant (*p* = 0.013, 95% CI), with a sensitivity of 87%.

When tested for 1-year mortality, the predictive performance was significant for CA 125 (*p* = 0.044, 95% CI) for a cutoff value of 103.8 U/mL and CA 125 + CEA (*p* = 0.030, 95% CI), even though CEA was not able to significantly predict mortality on its own (*p* = 0.21, 95% CI*).*

Positive predictive value (PPV) and negative predictive value (NPV) were calculated for the statistically significant TMs and are shown on Table 3. Although with neglectable PPV regarding both outcomes, CEA and CA 125 had NPVs of 87.9% and 93.1% in regard to PC, respectively. Also, NPV of 88.9% and 89.2% were met concerning mortality, for the same TMs. The combined performance of both TMs resulted in a NPV of 92.8% and 88.7% for PC and mortality, respectively.

## Discussion

Gastric cancer remains one of the deadliest cancers worldwide, mainly due to its nonspecific clinical presentation and advanced stage at diagnosis [4]. The improvement of surgical technique and tailored systemic treatment increased overall survival and is presently considered the only potentially curative treatment [1, 3]. Disease-free survival (DFS) in locally advanced GC, estimated to be 30–60%, confirms that a high percentage of patients treated with curative intent end up with recurrence of disease [9]. A key step to achieve long-term survival seems to be related to early diagnosis, given the aggressiveness of GC. This fact has been confirmed by the improved survival in Asian countries where population screening is implemented [4]. Attempting to find a cheap, practical and non-invasive method of screening for poor outcome at the time of diagnosis would allow to select patients who might benefit from a more aggressive, or different, type of treatment [13, 16, 17].

Some studies have tried to establish a relationship between serum and ascitic TMs and their role in different settings. They have been compared in terms of discrimination of benign and malignant causes of ascites, and their usefulness as predictors of the course of disease after curative surgery [11, 13, 18]. Shibata et al. reported that after curative surgery, when comparing CEA and CA 19.9, serum CA 19.9 showed a higher predictive value for recurrence of disease [9]. When tested in ascites, Du et al. found that CEA, CA 15.3, and CA 19.9 predicted PC with 94.6% accuracy [12]. Their isolated value could also be optimized when used in combination according to other series [11].

In the presented study, the populations’ demography followed the global epidemiologic data on GC, with an increased incidence of 65.8% in male patients and a median age at diagnosis of 70 years. In western countries with no screening, GC is a disease of the elderly, with more than 90% of the diagnosis being made in patients with over 55 years of age and in advanced stages of disease, consistent with our own data [19].

When checked for PC and mortality after 1 year, cT status proved to correlate significantly (*p* = 0.032), thus emphasizing the importance of an early diagnosis in order to increase DFS[4]. cN status on the other hand did not significantly correlate with either outcome (*p* = 0.177 for PC; *p* = 0.239 for mortality).

At the time of SL, serum and peritoneal TMs were measured. A significant correlation between both samples of the same TM was found for CA 19.9 and CA 72.4. However, the serum and peritoneal concentration of the other TMs did not correlate significantly which makes the peritoneal washing analysis not replaceable by the serum assay. To date, there have been conflicting data regarding this relationship, given that most of the studies were conducted with small population sizes. Tuzun et al. managed to correlate significantly serum and peritoneal TMs in patients with malignant ascites [18]. On the other hand, another comparative study concluded that peritoneal TMs were of increased value in terms of sensitivity in determining malignant ascites [11].

A ROC curve analysis was conducted in order to determine the predictive power of peritoneal TM assays. According to our data, peritoneal CA 125 above 107.6 U/mL has high yield for the prediction of PC (*p* = 0.019). Also, CEA was able to independently predict CP when above 2.0 ng/mL (*p* = 0.020). In similar studies, Yang et al. measured CEA in GC-associated malignant ascites and concluded that for values above 2.3 ng/mL, it had diagnostic value for malignant *vs.* benign ascites [16]. Another study by Taobo et al. concluded that serum CA 125 was significantly higher in patients with peritoneal metastasis in GC and that its measurement is useful in predicting curability [3]. Moreover, serum CEA has also been proven to be an independent risk factor for poor prognosis [20]. CA125, although primarily used in ovarian cancer, is frequently positive in cases of peritoneal recurrence, thus being considered an independent predictor of poor outcome [3].

Regarding PC, positive, and negative predictive values were calculated and, although both TMs had low PPVs (CEA: 52.8%, CA 125: 53.4, CA 125 + CEA: 52.7%), the NPV was significant for both, thus establishing that below the determined cutoffs, a GC patient is not likely to have disease progression in the form of PC within a year of the SL. In this setting, combining both TMs could provide a higher yield for a positive outcome (NPV of CA 125 + CEA: 92.8%).

Choosing to associate the peritoneal washing harvest for TM measurement to the cytology during SL makes this analysis cost effective, reproducible, and does not add any other invasive procedure to the patients’ treatment. In fact, doing so before starting any type of treatment could be another useful tool to select patients with a predictable, more aggressive, course of disease, and potentially tailor treatment options such as extending indications for hyperthermic intraperitoneal chemotherapy to patients with negative peritoneal washing cytology.

The presented study has, however, some limitations. Although widely used for the follow-up of digestive tract malignancies, CEA production varies according to cancer location, histologic subtypes in different disease stages and its concentration can be influenced by non-malignant conditions and inflammation-inducing external factors such as smoking [21]. Despite the statistically significant predictive value obtained, tumor markers have low diagnostic yield, and sensitivity. CEA and CA125 are not gastric cancer-specific, nor have a linear variation with PC, which can limit their widespread use as a predictive tool. This is supported by the fact that not all patients who developed PC after one year had TM measurements above the upper limit of the reference range at the time of the staging laparoscopy. Nevertheless, their positive predictive value in the peritoneal washing reached 70% in similar studies, which in turn makes them useful when above the determined threshold [16]. The analysis was made with a pool of patients of a single center and, due to the aggressiveness of GC and late diagnosis, the study’s population was reduced. Secondly, the patients were not all submitted to the same systemic treatment and the response to treatment was not taken into account. Also, some of the patients had disease progression and were not submitted to curative surgery. Further studies with larger sample sizes should be conducted to provide further information on TMs and their predictive value.

## Conclusion

CA 125 and CEA, individually and in association, for a cutoff value of 107.6 U/mL and 2.0 ng/mL, respectively, could be used to predict progression of disease and mortality within a year of the staging laparoscopy in GC patients. The same TMs have high NPV, making them a useful tool with high yield for both favorable and poor outcomes after treatment.

## Data Availability

The data that support the findings of this study are available from the corresponding author, João Luís Pinheiro, upon reasonable request.
